# Peripheral focused ultrasound stimulation and its applications: From therapeutics to human–computer interaction

**DOI:** 10.3389/fnins.2023.1115946

**Published:** 2023-04-14

**Authors:** Shi-Chun Bao, Fei Li, Yang Xiao, Lili Niu, Hairong Zheng

**Affiliations:** ^1^National Innovation Center for Advanced Medical Devices, Shenzhen, China; ^2^Paul C. Lauterbur Research Center for Biomedical Imaging, Shenzhen Institute of Advanced Technology, Chinese Academy of Sciences, Shenzhen, China

**Keywords:** peripheral focused ultrasound stimulation, peripheral nervous system, therapeutics, neuromodulation, acoustic radiation force, mid-air haptics, human–computer interaction

## Abstract

Peripheral focused ultrasound stimulation (pFUS) has gained increasing attention in the past few decades, because it can be delivered to peripheral nerves, neural endings, or sub-organs. With different stimulation parameters, ultrasound stimulation could induce different modulation effects. Depending on the transmission medium, pFUS can be classified as body-coupled US stimulation, commonly used for therapeutics or neuromodulation, or as an air-coupled contactless US haptic system, which provides sensory inputs and allows distinct human-computer interaction paradigms. Despite growing interest in pFUS, the underlying working mechanisms remain only partially understood, and many applications are still in their infancy. This review focused on existing applications, working mechanisms, the latest progress, and future directions of pFUS. In terms of therapeutics, large-sample randomized clinical trials in humans are needed to translate these state of art techniques into treatments for specific diseases. The airborne US for human-computer interaction is still in its preliminary stage, but further efforts in task-oriented US applications might provide a promising interaction tool soon.

## 1. Introduction

Ultrasound (US) waves are acoustic waves of frequencies above 20 kHz, higher than the upper audible limit of human hearing. Based on the biological mechanisms of three US effects (wave, mechanical and thermal effects), the US has been extensively used in various human-oriented applications, including diagnostics, surgery, therapy, and human-computer interaction (HCI; Zheng et al., 2015). Specifically, focused US (FUS) stimulation can be applied on either the central nervous system or peripheral extremities, similar to electrical stimulation modalities which are the most widely used. However, electrical stimulation is limited by its low spatial resolution and difficulty in modulating deep neural structures. In contrast, FUS can provide non-contact delivery of acoustic energy to a target with high spatial and temporal resolution, allowing for precise and localized stimulation of individual nerves, receptors, or other neuronal structures without affecting neighboring tissues.

Ultrasound intensity (W/cm^2^; power transferred per unit area) can be defined as the average intensity of an individual pulse (spatial-peak pulse-average, I_SPPA_) or with the total time-averaged intensity (spatial-peak-temporal-average, I_SPTA_), with the latter one being more commonly used in US stimulation. The intensity of US stimulation attenuates exponentially with the propagation distance due to absorption, reflection, and scattering in heterogeneous media. Other typical parameters include acoustic pressure, frequency, mechanical index, sonication duration (SD), duty cycle (DC), pulse duration (PD), and pulse repetition frequency (PRF). The central frequency of the US transducer is frequently used as the US stimulation frequency ranging from 20 kHz up to 10 MHz, and the sonication duration ranges from milliseconds to hundreds of seconds, or even longer, depending on the specific task requirements. For example, air-coupled US applications typically have frequencies of 40–70 kHz, while body-coupled US applications usually range from 0.2 to 5 MHz. Higher-frequency US stimulation modalities provide higher spatial resolution, but also experience rapid attenuations. Theoretically, 1 and 5 MHz pFUS can result in spatial resolution close to their half-wavelengths, which are 0.75 and 0.15 mm in the human body, respectively. The acoustic pressure ranges from tens of kilo-to mega-pascals. The mechanical index characterizing the cavitation is defined as the peak negative acoustic pressure (MPa) divided by the square root of frequency (MHz). In US neuromodulation, the duty cycle, which is the ratio between the pulse width and the pulse repetition period, can be as high as 100% (Szabo, 2013).

Unlike diagnostic US imaging which employs relatively low-intensity US of 0.05–0.5 W/cm^2^ (Chen et al., 2020), the intensity of FUS can vary widely. Both low-intensity and high-intensity US have been utilized through thermal effects or non-thermal effects. Different US stimulation parameters could induce diverse effects. By applying a US pulsed wave on the target, low-intensity focused ultrasound (LIFU, 0.5–100 W/cm^2^) could induce mechanical effects of acoustic radiation force (ARF) or other biological mechanisms, and such effects are generally reversible and not harmful to the organs (Zheng et al., 2015). Low-threshold mechanoreceptors, such as tactile receptors and auditory nerve endings, may also be activated. In contrast, high-intensity focused ultrasound (HIFU; > 100 W/cm^2^) with continuous US waves can induce tissue heating, and the thermal effects could be used for therapeutic ablations (Martin et al., 2009). Furthermore, activation of vibrotactile and pain sensations needs HIFU-level peripheral stimulation (Lee et al., 2014).

The mammalian nervous systems are composed of the central and peripheral nervous systems (CNS, PNS). The CNS includes the spinal cord and the brain, regulating the responses of the entire body. In contrast, the PNS consists of all the nerves outside of the brain and spinal cord and often directly influences peripheral functions. The PNS provides the CNS information about the external and internal environment by sending afferent sensory information to the brain and efferent neural signals to peripheral organs to tune physiological outputs (Kandel et al., 2012). Furthermore, it could be classified as somatic PNS, which includes the sensory neurons that receive information from the skin, muscles, and joints, and autonomic PNS, which modulates involuntary functions such as the heart and smooth muscles in the gut and glands. Because the nervous systems are complex functional networks, peripheral diseases or dysfunctions may be challenging to determine the precise neural circuits or targets in the CNS, such as the widespread pain circuitry in the brain. PNS stimulation could directly modulate peripheral functions such as chronic pain, preventing the off-target effects of CNS stimulation. Although the FDA has approved peripheral electrical stimulation devices for various sensorimotor dysfunctions (Johnson and Wilson, 2018), most electrical stimulation devices were based on invasive electrodes and induced surgical risks. Non-invasive US strategies may be an alternative for treating peripheral dysfunctions, but peripheral US stimulation have received less attention than central US stimulation. Understanding the fundamental mechanisms underlying US stimulation could undoubtedly guide US parameter selections and intervention designs, and it might enable more robust and targeted practical applications.

**Figure 1:**
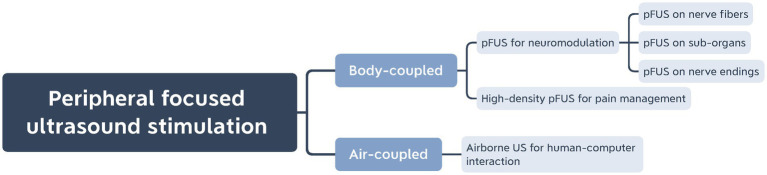
General structure of the review.

This paper focuses on the mechanisms and applications of peripheral focused US (pFUS) stimulation. However, applications such as bone healing and soft tissue regeneration are not covered (Best et al., 2016; Harrison and Alt, 2021). Unfocused stimulation or ultrasonic neuromodulation of the CNS is not the scope of this article, which has been reviewed in many previous summary papers (Naor et al., 2016; Blackmore et al., 2019; Kim T. et al., 2021). The intensities of pFUS stimulation are generally higher than that of central US stimulation, and the stimulation target could be peripheral neuroreceptors, nerve fibers, or sub-organs. Depending on the medium, whether the ultrasound transducers are in close contact with human skin or other organs, pFUS could be classified as body-coupled contact US stimulation, often used for therapeutics or neuromodulation, or air-coupled contactless US haptic system, which allows diverse HCI paradigms. However, the underlying mechanisms of pFUS remain unclear, and human-oriented applications are still under development. This review provides a comprehensive overview of different peripheral ultrasound stimulation applications summarizing the published studies and forecasting future trends (Figure 1).

## 2. Body-coupled peripheral focused ultrasound stimulation

**Figure 2:**
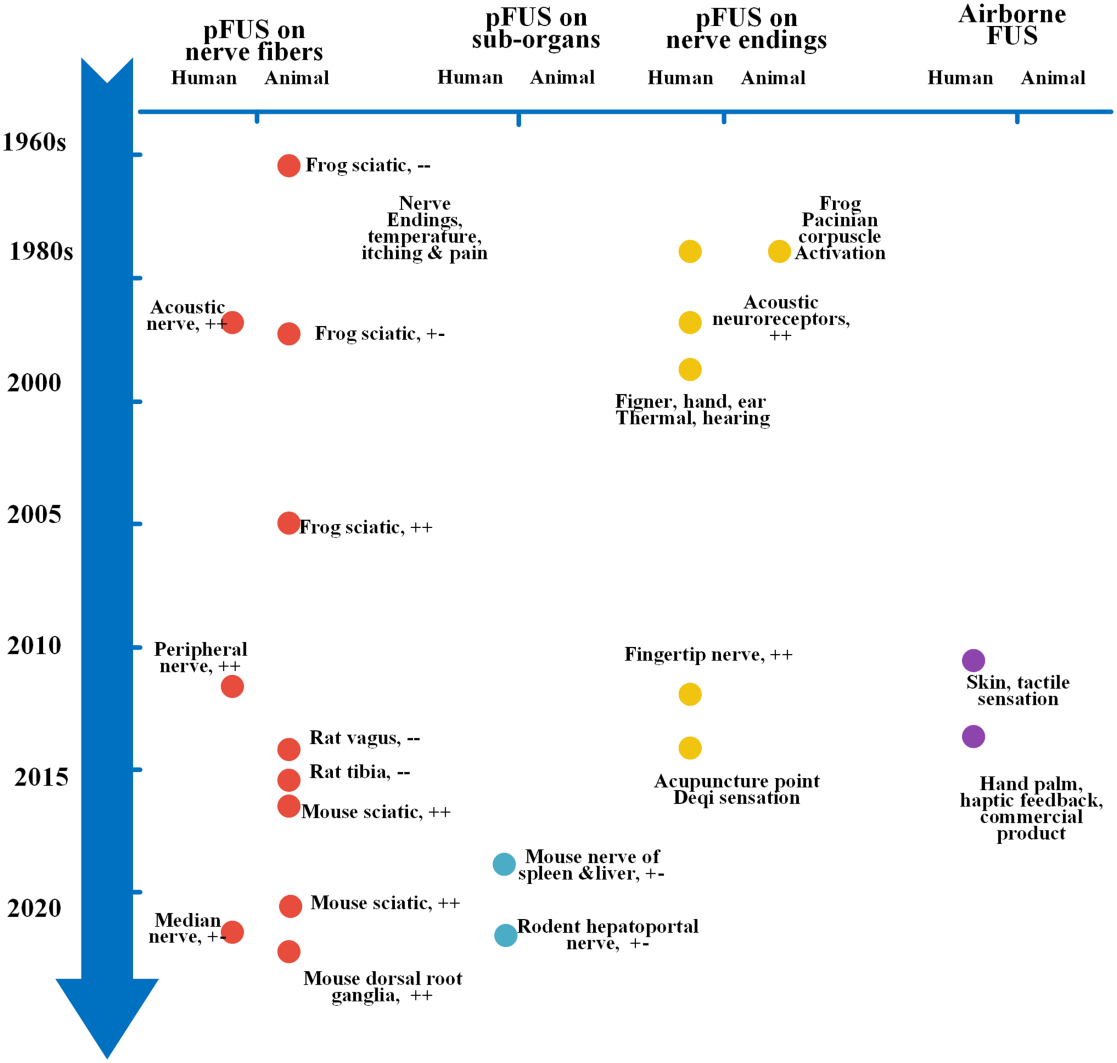
Timeline and brief history of representative pFUS applications. Each dot represents one typical finding, different color indicates different stimulation applications, left side for animal studies, right side for human studies. *y*-axis, year in sequence, before 2000, each tick means 20  years, after 2000, each tick means 5  years; *x*-axis, different pFUS techniques. pFUS, peripheral focused ultrasound stimulation. ++, excitation; +−, modulation or mixed effects; −–, inhibition.

Medical use of ultrasound imaging started in the early 20th century, and it was initially used to diagnose peripheral muscular disorders and rheumatoid arthritis or to guide neurosurgery or other therapeutics. A variety of mechanisms can explain the biological effects of therapeutic US (Miller et al., 2012). FUS has been utilized in medical ablation since the 1940s for its thermal effects, such as treating tumors, kidneys, essential tremors, and bladder. However, such effects may be irreversible (Ghanouni et al., 2015). FUS has been used for creating local anesthesia and revolutionized chronic pain management by blocking related neural functions through its thermal effects (di Biase et al., 2021). Some studies proposed non-thermal acoustic radiational effects of US as the working mechanism for low-intensity pFUS (Downs et al., 2018; Lee et al., 2020), while others proposed intramembrane cavitation as the working mechanism for general ultrasonic neuromodulation (Plaksin et al., 2016). Several hypotheses were proposed to explain the neuromodulation effects of FUS (Kamimura et al., 2020). Additionally, high-intensity US stimulation of rat sciatic nerves could induce inertia cavitation with strong acoustic forces (Lee et al., 2015a). Understanding these underlying working mechanisms would allow for more reliable and targeted interventions. This section summarizes the pre-clinical and clinical trials of both high and low intensities. Figure 2 indicates a gradual increase in interest in the neurostimulation effects of pFUS. Both animal and clinical studies have shown intriguing results for high-intensity peripheral pFUS in pain management. Additional clinical applications are still in the early stages and translational investigations are on the way.

### 2.1. Peripheral focused ultrasound for neuromodulation

Early studies in the 1950s demonstrated that the US could reversibly inhibit neuronal functions of the CNS in animals (Fry et al., 1958). Transcranial focused ultrasound (tFUS) has been shown to provide non-invasive neuromodulation of deep brain tissue in animals and humans over the past two decades, and it could work as a promising therapeutic tool (Tyler et al., 2008; Tufail et al., 2011). Compared to tFUS, the fundamental mechanisms of pFUS neuromodulation are only partially understood and even under debate, and its clinical applications are still in the experimental stage. Currently, the most common application of low-intensity pFUS is on the peripheral nerve fibers, sub-organs, and nerve endings, with varying stimulation effects depending on the specific parameter settings. The underlying mechanisms of pFUS are also remain diverse and still being studied. This subsection first discussed the metrics for assessing neural functions after pFUS. Afterwards, we summarized the development history and working mechanisms of pFUS applications in different target types. Finally, we provided a tentative prospect for the future directions of pFUS.

#### 2.1.1. Metrics for assessing neural functions after pFUS

Quantitative evaluations of stimulation effects are essential for understanding the working mechanisms of pFUS. Although US is compatible with a wide range of neurophysiological measurement tools, it is challenging to capture immediate peripheral neuronal response to US stimulation due to the protective soft connective tissues surrounding mammalian peripheral nerve axons, making it difficult to complete electrophysiological measurement for individual nerve axons. In contrast, while neurons in the CNS are not protected by such tissues and can be reliably recorded using invasive electrodes placed within the skull (Kandel et al., 2012). Single-unit recording can measure temporal information of spike signals with dominant action potential propagation (Chen et al., 2017), but its application remained limited in pFUS applications due to technical difficulty. Only one study reported that pulsed pFUS increased peripheral nerve conduction velocity, as measured by single-unit recordings (Ilham et al., 2018). Compound action potentials (CAPs) are the summation of action potentials from multiple muscle fibers (Merletti and Farina, 2016). Previous studies have utilized CAPs as a primary neurophysiological metric for assessing peripheral neural activities after pFUS (Lee et al., 2015b, 2020; Wright et al., 2017; Downs et al., 2018). However, various parameters easily influence CAP properties, such as recording positions and electrode properties, making it sophisticated to evaluate peripheral nerve functions after pFUS accurately.

The biomedical community has investigated various methods to investigate the effects of pFUS on the peripheral nervous system. Early studies also explored indirect measures such as refractory periods, muscle force, sensory action potentials and image-based techniques. For instance, a study demonstrated that 250 kHz focused US could suppress rhythmic bladder contractions in ten anesthetized rats, with longer latencies and refractory periods than conventional electrical stimulation, indicating a potential outpatient treatment for overactive bladder (Casella et al., 2017). Electroencephalography (EEG) and functional magnetic resonance imaging (MRI) analysis also revealed that pulsed FUS on the periphery could elicit a representative somatosensory response, as evidenced by evoked potentials and blood oxygen level-dependent responses (Legon et al., 2012). Another study found that pFUS on the sciatic nerves could induce EMG responses similar to that of conventional electrical stimulation in mice without harming the nerves or surrounding regions (Downs et al., 2018). Most prior FUS targeting modalities required MRI or B-mode US imaging to guide the treatment (Wu et al., 2005; Weeks et al., 2012). Notably, B-mode US imaging provided safe, accurate, and convenient real-time monitoring and targeting of peripheral nerve disorders (Carroll and Simon, 2020). For example, a US image-guided focused US study exhibited safe and controllable modulation of evoked motor neuron activity *in vivo* with PRF of less than 40 Hz by facilitating motor neuronal response on electrically evoked potential, while PRF more than 100 Hz could induce inhibition effects with irreversible temperature elevation of above 15°C (Kim et al., 2020). It was shown that US efficacy depended on PRF or duty cycle in well-characterized nervous system of Caenorhabditis elegans nematodes (Kubanek et al., 2018). Additionally, US-based displacement imaging illustrated a correlation between acoustic radiational forces and motor activations *in vivo* peripheral nerves during 4 MHz pFUS in mice, with EMG-based CAPs increasing with interframe nerve displacement (Lee et al., 2020). Moreover, pFUS could induce representative neurogenic axon reflexes of peripheral nerves in the mouse sciatic nerve, as evidenced by distinct blood flow changes after pFUS (Kim M. G. et al., 2021).

In summary, metrics to assess neural functions after pFUS are intriguing and under development. Further research based on these metrics is needed to fully understand the underlying mechanisms of various pFUS applications.

#### 2.1.2. Peripheral focused ultrasound stimulation on nerve fibers

In Harvey (1929), a pioneering study reported that ultrasonic stimuli could cause rhythmic contractions of quiescent ventricular muscles in frogs and turtles. Subsequently, peripheral US stimulation has been tested *in vivo*, *in vitro*, and *ex vivo* animal models. By increasing the level of brain-derived neurotropic factor, unfocused pulsed ultrasound could facilitate nerve regeneration in a rat sciatic nerve crushed injury model (Raso et al., 2005; Jiang et al., 2016; Ni et al., 2017). The effects of the pFUS can vary depending on the stimulation parameters and may involve either mechanical or thermal effects. pFUS has been shown to inhibit and promote neural activities in various mammalian and invertebrate nerves, such as frog sciatic nerves, crab leg nerves, rat vagus and tibial nerves, mouse sciatic nerve, and human median and sciatic nerves (Young and Henneman, 1961; Lele, 1963; Mihran et al., 1990; Tsui et al., 2005; Dickey et al., 2012; Juan et al., 2014; Casella et al., 2017; Downs et al., 2018). Spike activities and conduction velocities are important early-stage metrics indicating that mammalian nerves respond differently to US stimulation. It was observed that the smallest C fibers are the most responsive while the largest A-α fibers are the least sensitive, and that the US may achieve neuromodulation through electro-mechanical resonance properties (Legon et al., 2012).

The effects of US stimulation are affected by many factors, including the duration, intensity and frequency of the stimulation. Long pulses (5 s–5 min) can induce thermal effects or mechanical effects depending on the duty cycle of the US stimulation. In contrast, short pulses of US stimulation may induce changes in compound action potentials (CAPs) with either enhancement or suppression *via* mechanical effects (Mihran et al., 1990). According to the US Food and Drug Administration (FDA), the intensity of diagnosis US imaging should be within 0.72 W/cm^2^ in I_SPTA_, US stimulation of I_SPTA_ below 1 W/cm^2^ is generally considered low-intensity, and transcranial focused ultrasound stimulation with such intensity could effectively introduce neuromodulation effects in both animals and humans (Tyler et al., 2018). However, whether US intensity < 1 W/cm^2^ could consistently modulate peripheral nerve functions in still controversial. A study reported that US intensity < 1 W/cm^2^ was insufficient for identifying significant neuromodulatory effects on the PNS *in vitro* (Ilham et al., 2018), while others showed US stimulation within FDA recommendations could elicit modulatory effects in the PNS (Kim et al., 2012; Downs et al., 2018; Riis and Kubanek, 2022). Regarding the working mechanism for pFUS, previous reviews summarized that pFUS with I_SPTA_ between 1 and 200 W/cm^2^ is not intense enough to elicit temperature-driven neuromodulation, and acoustic radiation force is likely the dominant effect for pFUS with I_SPTA_ less than 200 W/cm^2^ (Feng et al., 2019). Most studies on body-coupled peripheral nerve stimulation utilized acoustic frequencies ranging from 0.25 to 7 MHz, with a spatial resolution of roughly 3 mm to 0.1 mm. The selection of a spatial resolution depends on the stimulation location under the skin and the size of the stimulation targets. Furthermore, there was still no recognized standard for parameter settings. Descriptions of US intensities used in different studies include I_SPPA_, I_SPTA_, pressure and so forth, making it difficult to compare distinct stimulation settings. Further investigations are needed to determine the optimal US parameter space for safe and efficient US stimulation protocols.

A recent mouse *ex vivo* study found that high-intensity, milli-seconds FUS pulses (350–500 W/cm^2^) can evoke action potentials for both myelinated A fibers and unmyelinated C fibers in mouse dorsal root ganglia, with millisecond latencies compared with electrical stimulation, implying PIEZO2 ion channel-mediated mechanisms (Hoffman et al., 2022). The study manifested that transcutaneous FUS stimulates peripheral nerve activity by activating intrinsic mechano-transduction mechanisms in neurons, and the modulation performance varied in different types of neurons (Hoffman, 2019). Furthermore, decreased FUS-evoked action potentials at higher FUS stimulus intensities may account for the activation of potassium channels after FUS, subsequent study should also evaluate the hypothesis thoroughly. However, the modulation effects of FUS might be limited for peripheral neurons which are lacking mechanosensitive ion channels (Hoffman et al., 2022). A recent study showed that US could not excite an isolated pig sciatic nerve *in vivo*, but it could reliably inhibit nerve functions across a wide range of parameters through a thermal effect (Guo et al., 2022). These findings contradict previous research on non-isolated nerves. It might be accounted for the lack of mechanosensitive ion channels in pig axon membranes. Another explanation of Guo’s paper is that the mechanical environment of the neuron might change in the extracellular matrix, altering the response of those ion channels following pFUS. Higher pFUS intensities indicate more severe safety challenges, and the safe parameter space was not sufficiently explored for practical applications. Moreover, mathematical modelling could also play important role in understanding the modulation effects of US stimulation. Several models have been proposed to explain the US neuromodulatory effects regarding radiation force and neuronal action potentials, but no consensus has been reached (Plaksin et al., 2016; Lemaire et al., 2019).

Beyond previous efforts in animal models, pFUS studies were also conducted in human beings. Preliminary studies showed that pFUS could induce representative suppression of somatosensory evoked potentials in human subjects, as measured by EEG (Kamimura et al., 2019). Further, US imaging-guided 1.1 MHz pFUS could modulate median nerve functions and change thermal pain perception in healthy human subjects, implying potential FUS clinical applications in pain management (Lee et al., 2021). Later clinical trials in patients were indispensable to assess the functional role of pFUS in treating specific pain syndromes.

#### 2.1.3. Peripheral focused ultrasound stimulation on sub-organs

In addition to sensorimotor functions of somatic PNS, US stimulation could also target autonomic PNS, such as the sub-organs which are innervated by nerve pathways. For instance, US stimulation prevented renal ischemia–reperfusion in mice by modulating of the splenic cholinergic anti-inflammatory pathway (Gigliotti et al., 2013). Further, spleen-targeted pFUS could modulate the cholinergic anti-inflammatory pathway and reduce cytokine (for example, TNF, IL-1β, and IL-6) response to endotoxin to the same levels as implanted vagus nerve electrical stimulation (Cotero et al., 2019a). Recent research has also demonstrated the effectiveness of spleen-focused US stimulation in the treatment of inflammatory diseases. Spleen-targeted pFUS could effectively improve the severity of arthritis in an arthritis mouse model by influencing CD8 + T cells (Hu et al., 2022). Single-cell RNA sequencing of splenocytes and experiments in genetically-immunodeficient mice models illustrated the functional role of T and B cells in the anti-inflammatory pathway (Zachs et al., 2019). In addition to the spleen, hepatic US stimulation could modulate pathways that regulate blood glucose and is as effective as vagus electrical stimulation in suppressing the hyperglycemic effect of endotoxin exposure (Cotero et al., 2019a). Several studies have manifested that selective activation of the hepatoportal nerve plexus *via* pFUS could improve glucose homoeostasis and glucose tolerance and utilization in diabetes rodent and swine models (Huerta et al., 2021; Cotero et al., 2022).

Beyond previous efforts, additional explorations are required to elucidate the modulation mechanism in human subjects and translate these state of art techniques into clinical practice for inflammatory and metabolic disorders. pFUS has the potential to be a promising bioelectronic medicine method as an alternative to conventional electrical stimulation modalities.

#### 2.1.4. Peripheral focused ultrasound stimulation on nerve endings

Sensorimotor functions can be encoded by neurosensory receptors and nerve endings beneath the epidermis. Unlike most pFUS studies on nerve fibers or sub-organs, which were in the pre-clinical stage, the US applications in peripheral nerve endings have been successfully conducted in human subjects. As early as 50 years ago, classical electrophysiological experiments in animals and humans showed that 1–3 MHz pFUS could activate skin mechanoreceptors and induce tactile sensations. Meanwhile, the stimulation of nerve mechanoreceptors such as Pacinian corpuscles can generate temperature and pain sensations (Gavrilov et al., 1976). Several pioneering studies by Soviet Union scientists have demonstrated that pFUS of short duration and relatively high intensity can induce various somatic sensations such as tactile, thermal, hearing, and pain without adjunct harm to surrounding tissues. The modulation effects might attribute to the mechanical effects of US stimulus (Gavrilov et al., 1996). The US intensity (1–200 W/cm^2^) can activate low-threshold mechanoreceptors in the skin, muscles, joints, and other typical components. As the stimulus intensity increases, the sensations progress from tactile to thermal and finally to pain perception. These sensations can be elicited by pulsed mode, amplitude-modulation or pulse-amplitude modulated pFUS. However, continuous US stimulation failed to elicit tactile sensations like pulsed stimulation. The most representative thermal sensations were found to occur at an intensity range of 10–30 W/cm^2^ (I_SPTA_), while vibrotactile and nociception could happen in the range of up to 100 W/cm^2^ (I_SPTA_; Lee et al., 2014). Furthermore, US on acupuncture points (LI4) can elicit deqi sensations, implying its potential medical applications (Yoo et al., 2014).

Moreover, a subsequent study manifested that the sensations induced by pFUS were related to the density of mechanoreceptors, and different neuroreceptors respond differently to the same US dosage (Dickey et al., 2012). It could explain that the sensations induced by pFUS differ in different parts of the human body and the sensations of fingers require lower US stimulation intensity than that of palms with the same stimulation frequency. Further, sensations appear to be more effectively triggered at lower frequencies of US stimulation (Gavrilov, 2016), which might account for increased radiational force and neuronal displacements after lower frequency US, and mechanoreceptors and ion channels could be better modulated (Coste et al., 2012). An underwater human study also determined that a 300 kHz stimulus is more effective than a high 900 kHz stimulus in stimulating excitable mechanoreceptors and nerve fibers in the human PNS, providing transducer selection guidelines for human peripheral nerve stimulation (Riis and Kubanek, 2022). The study excluded the effects of heating and cavitation from their experimental results, and future studies are required to determine the functional role of US in peripheral neural receptors.

Peripheral focused ultrasound stimulation on peripheral nerve endings could diagnose various neurological, dermatological and hearing disorders (Gavrilov and Tsirulnikov, 2012). For example, neurological patients have higher tactile thresholds after pFUS than healthy controls, and this pattern could be used to diagnose of neurological diseases (Gavrilov, 1984). Similarly, pFUS could diagnose hearing disorders by comparing standard threshold ultrasonic audiograms in healthy controls to audiograms measured by pFUS in hearing diseases (Tsirulnikov et al., 1988). However, the potential risks of prolonged US exposure are non-negligible and worth further explorations. Due to high-frequency US transmission problems in the air and inherent safety challenges for surrounding organs, pFUS applications on nerve endings remain limited. Furthermore, optimized and appropriate stimulation settings for diverse applications are not well characterized for diverse applications, and a wide range of acoustic parameters may influence peripheral neuroreceptors’ responses after stimulation.

#### 2.1.5. Future directions of pFUS

To further improve the neuromodulatory effects of pFUS and facilitate practical applications, the transducer designs, measurement, and guidance tools are among the primary technical challenges. Most previous studies utilized single-element transducers with a fixed focal point, poor spatial resolution, limited transducer bandwidth, and no image guidance (Gao et al., 2019; Luo et al., 2020). Current US stimulation systems cannot satisfy the all-around requirements of stimulation targets. To address these limitations, the development of dual-mode 2D transducer arrays allow for simultaneous recording and neuromodulation (Zhang et al., 2021), and multifrequency transducers may also contribute to more practical pFUS applications (Constans et al., 2017). Additionally, acoustic metamaterials can manipulate and control sound waves more effectively than conventional materials, allowing for more focused and reliable sound manipulations (Li et al., 2014; Cummer et al., 2016). Next-generation US transducers, such as capacitive or piezoelectric micromachined ultrasonic transducers, could be manufactured with flexible and wearable materials to produce broadband transducers (Khuri-Yakub and Oralkan, 2011). However, more efforts are necessary to evaluate these new peripheral ultrasonic neuromodulation techniques in real-world applications. Looking forward, we envision that US stimulation has the potential to serve as a new type of bioelectronic medicine, offering an alternative to traditional pharmaceutical treatments (Cotero et al., 2019b).

### 2.2. High-intensity pFUS for pain management

Pain is an unpleasant sensory and emotional experience associated with, or resembling that associated with actual or potential tissue damage (Walker et al., 2021). According to the International Association for the Study of Pain (IASP), chronic pain is defined as pain lasting longer than 3 months (Ashburn and Staats, 1999). Notably, chronic pain could be categorized into three types of damage or disease: nociceptive pain of direct tissue, neuropathic pain of somatosensory system, or mixed pain of both. When conventional treatments fail to relieve painful symptoms, temporary or permanent disruptions of functional pathways may be necessary for pain relief. Compared to other minimally-invasive or invasive nerve block techniques, HIFU-based pain management could reduce the risk of invasive surgery due to its non-invasive nature. HIFU could be targeted on both the central and peripheral nervous systems. Unlike central stimulation, which targets on specific neural pathways or regions in the brain or spinal cord, peripheral HIFU delivers local heating on peripheral nerves or regions *via* either ablation or reversible inhibition effects. Temperature elevation is the dominant mechanism of nerve conduction inhibition by HIFU (Lele, 1963), and non-thermal effects are not clear (Colucci et al., 2009). Other studies also reported that HIFU can induce mechanical effects on the giant axon of live earthworms (Wahab et al., 2012).

The early FUS investigations in the 1960s indicated that FUS effects were temperature dependent, with minor temperature differences between ablation and reversible effects (Lele, 1963). FUS was found to modulate C fibers without affecting A fibers, and reversible effects on conduction were observed after 0.4–1.0 s of US exposure in *ex vivo* frog sciatic nerves (Young and Henneman, 1961). Another study revealed that FUS stimulus on the optic nerve induced partial or total inhibition of visually evoked potentials for 4–5 min (Adrianov et al., 1984). Additionally, 30 s continuous wave sonication of HIFU could inhibit nerve conduction and provide a complete and temporary conduction block of normal bullfrog sciatic nerves (Colucci et al., 2009). In contrast, US-imaging-guided peripheral HIFU can only partially and temporarily block the CAPs of sciatic nerves in rat plantar foot muscles, and histological evidence of axonal demyelination and necrosis of Schwann cells or axons lesioning was reported (Foley et al., 2007, 2008). A recent study showed that some CAP parameters are altered similarly by HIFU and local anesthetics in an *ex vivo* rat sciatic nerve model, with minor but significant differences (Anderson et al., 2022). Overall, FUS has been studied for its potential in pain management, but more research is needed to fully understand the effects and applications of HIFU in pain management.

Most prior pre-clinical studies have stimulated sensory and motor nerves together, and thus it is necessary to distinguish the functional roles of high-intensity pFUS in motor or sensory nerves separately. *In vitro* experiments showed that CAPs of the sciatic nerve and sensory action potentials of the sural nerve were temporarily and incompletely blocked by HIFU and fully recovered after pFUS with appropriate stimulation parameters in normal and diabetic rats. The blocking effects lasted 10 to 30 min. These results indicated that high-intensity pFUS could safely and reversibly suppress nerve conduction in diabetic rats for analgesic applications, implying application in blocking sensory nerves reversibly and providing peripheral pain relief in diabetic rats (Lee et al., 2015b). Earlier research examined the relationship between thermal doses and changes in peripheral nerve histology and neural functions (Vujaskovic et al., 1994), and it appears that both safety and intervention efficiency can be achieved. Patients with vocal cord paresis after single-session HIFU of thyroid nodules could recover within 6 weeks, suggesting that thermal power did not induce irreversible nerve damage in these cases (Lang et al., 2017). Further, medial branch nerves could be directly ablated with MRgHIFU in swine models, effective nerve thermal necrosis was created without damaging adjacent tissues (Kaye et al., 2016). Similarly, HIFU treatment on the occipital nerves was investigated in a validated rodent headache model of chronic migraine, pulsed HIFU but not ablative HIFU enhanced mechanical thresholds post-therapy like sumatriptan at day three after treatment in the periorbital region, suggesting the potential of pulsed pFUS in treating migraine (Walling et al., 2018).

To guarantee safety during high-intensity pFUS treatment, it is necessary to monitor changes in peripheral nerves during treatment. While there was minimal evidence that HIFU-induced heat induces adverse effects of on peripheral structures, US guidance allows for target-specific thermal energy deposition to the peripheral nerve, making US-guided HIFU for nerve ablation possible (Worthington et al., 2016). Furthermore, MRI thermometry could non-invasively detect body temperature and monitor organic changes following pFUS (Jiang et al., 2020). In a cadaveric and laboratory feasibility study, MRI-guided FUS (MRgHIFU) illustrated temperature elevations of the trigeminal nerve using a gradient echo-sequence (Monteith et al., 2013). Another pilot pig study showed that MRgHIFU with 3D MR neurography guidance could be used for targeted peripheral nerve ablation, such a system could be applicable for post-treatment thermal tracking without contrast injection (Huisman et al., 2015a). Diffusion-weighted imaging and tractography can also effectively visualize target peripheral nerve segments and assess the microstructural changes after MRgHIFU (Walker et al., 2021). Nevertheless, the spatial resolution of imaging techniques such as US and MRI may still be limited for peripheral nerves such as sciatic nerve with a millimeter lever or even smaller, highlighting the need for future advancements in this area.

Though the underlying mechanisms of pFUS-based pain management have not been thoroughly investigated, preliminary clinical applications have been already performed in typical pain symptoms. With CE and FDA approval, pFUS has been applied in human pain relief for uterine fibroids, bone metastases (Huisman et al., 2015b), and low back pain due to facet joint osteoarthritis (di Biase et al., 2021). Furthermore, pFUS could alleviate pain in cancer patients by inducing tissue denervation, tumor mass reduction, and neuromodulation, all of which can influence pain-related neural pathways (Dababou et al., 2018). It outperforms conventional analgesic therapies because of its non-invasiveness, rapid pain control, safe repetition, and easy combination with chemotherapy or radiation therapy. A phase I single-arm clinical trial indicated that HIFU on the nerve terminals of the facet joints could reduce pain and improve functional abilities in patients with facet joint arthritis (Weeks et al., 2012). Phantom limb pain was also a significant concern, potentially influencing the life quality of almost half of the amputee patients. An exploratory case study exhibited MRgHIFU-mediated ablation of stump neuromas could reduce pain intensity in patients with postamputation neuropathic pain (Nachtigal et al., 2022). The cause of such postamputation neuropathic pain is complex, stemming from both the CNS, the spinal cord and peripheral nerves. Therefore, more research is required regarding the mechanism, safety and long-term efficacy. Further randomized controlled clinical trials with large sample sizes measures are necessary to determine the clinical efficacy of the latest HIFU-based pain management strategies.

## 3. Airborne ultrasound stimulation

### 3.1. Physics and mechanisms

Sensing is a fundamental process for human awareness of both the self and the environment. It could collect and transfer external information from the nervous system to the brain. The sense of touch or haptics is a critical part of the somatosensory system, with most touch-related afferent nerve fibers distributed in the human skin and internal organs. Typical neuroreceptors include the mechanosensory receptors, cold and warm thermoreceptors in the skin and kinesthetic inputs from the muscles, tendons, and joints. To generate tactile sensation, tactile information from peripheral cutaneous receptors is transferred *via* the dorsal column nuclei to the thalamic nuclei and then to the somatosensory cortex (Gallace and Spence, 2010).

Human-computer interaction (HCI) involves the interaction between a user with a machine by physical, cognitive, and affective aspects. Physical interaction techniques include vision, audition, and touch (Karray et al., 2008). Haptic devices allow HCI by producing artificial touch sensations, which could be classified as contact or contactless systems. Contact haptic devices that require user-device contact, such as static tangible artefacts, force feedback devices, and shape-changing tangible user interfaces (Hajas, 2021). Contactless airborne or mid-air haptic devices enable tactile sensations without touching or visual guidance, allowing free movement and combination with other techniques. Among airborne systems, US-based acoustic radiation pressure could provide focused US output on peripheral neuronal structures and receptors, remotely inducing spatially or temporally patterned vibrotactile sensations on the human skin. This technique ignited widespread research and industry interest (Hoshi et al., 2010; Carter et al., 2013). Compared with other contactless approaches like air-jet, lasers, electric arcs, and electromagnetic fields, ultrasonic haptics has advantages that allow multi-points and complex stimulation, fine-grained spatial and temporal resolution, and real-time interactions across large 3D workspaces (Rakkolainen et al., 2021).

Unlike body-coupled therapeutic pFUS systems, airborne focused ultrasound haptics devices require hundreds of waves emitted from air-coupled phase US arrays to induce one or multiple focal points with enhanced ultrasonic amplitude. It has already been applied in human-oriented applications without notable safety concerns. These devices use dynamic phase array control techniques to create a focal point of 20 mm above the middle of the transducer array and can fulfill complex haptic patterns as required (Iwamoto et al., 2008). Ultrahaptics is a typical haptic feedback system that uses a phased array of US transmitters above an interaction surface to provide multi-point haptic feedback. It was commercialized by Ultraleap Limited in 2013 (former Ultrahaptics; Carter et al., 2013). The system was integrated with the Leap Motion controller, which could provide real-time tracking of human postures with high speed and accuracy. The influence of human hands on US transmission was not considered in most existing mid-air US systems, which could be addressed by integrating sound field synthesis and reflection (Inoue et al., 2016). Most mid-air haptic devices can utilize 40 kHz US with an 8.5 mm wavelength, which can meet most haptic sensation requirements and has acceptable power consumption and acoustic attenuation. However, the resolution is inherently inadequate when compared to body-coupled MHz-level FUS (Rakkolainen et al., 2021). The absolute threshold of distinguishable ultrasonic tactile stimulation can be estimated by psychophysical experiments (Jones and Tan, 2013; Raza et al., 2020). The minimum perceivable acoustic radiation force of focal points produced by 40 kHz ultrasound arrays was larger than that produced by 70 kHz (Ito et al., 2016). A series of psychophysical experiments were carried out to estimate the absolute threshold of perceivable ultrasonic tactile feedback (Raza et al., 2020). Though higher frequency indicates higher spatial resolution for US stimulation, it is rarely utilized in mid-air haptics due to power dissipation and acoustic transmission problems. Recent advances in transducer designs, such as soft printed polymer transducers, are in development but are not yet ready for commercial use (van Neer et al., 2019). The strength of airborne haptics and the effect ranges remain insufficient when compared with physical hardware buttons, which would hamper its practical usage. Large-scale arrays could expedite haptic feedback in wider interaction areas and more applicable scenarios (Suzuki et al., 2019). Moreover, delay control strategies are critical when simultaneously controlling hundreds or thousands of transducers (Chen et al., 2020). Indeed, due to acoustic transmission law, the airborne US can only work in homogeneous media, which might limit its applications in some special conditions.

Previous studies have focused on touchless stimulation to elicit tactile sensations by advancing hardware or rendering tactile patterns for perception, both spatial and temporal properties are the main research interests. Because human tactile perception cannot directly receive high-frequency US stimulation beyond the normal vibrotactile perception range (5–1,000 Hz; Kandel et al., 2012), the airborne US haptics system requires focal point modulation to stimulate mechanoreceptors. Different techniques were proposed to improve the perception of focal points and increase tactile perceptions, including amplitude modulation at 200 Hz (Palovuori et al., 2014), lateral modulation with focal points repeatedly moving across target positions (Takahashi et al., 2018), and spatio-temporal modulation with focal points moving along an arbitrary trajectory of any shape and size (Frier et al., 2018).

Moreover, ultrasonic mid-air haptics could provide textured graphics by using a haptic mapping function (Pittera et al., 2019). Recent studies fulfilled haptic rendering of 2D geometric shapes with a dynamic tactile pointer, effectively increasing the shape identification accuracy effectively (Hajas et al., 2020). Existing mid-air haptic devices are still struggling to build complex tactile perceptions. The sensation of motion, shape of object, textural surfaces, and abstract dynamic patterns are among the research and commercial concerns for further development, please see the existing review paper more rendering details (Rakkolainen et al., 2021). Considering the safety of high-intensity focused airborne US, the radiation forces and focal sound pressure of a US-based haptic feedback device on a user’s hand were measured with a microphone and a balance, respectively. Such measurements could facilitate more precise localization of the mid-air haptics system (Liebler et al., 2020). Different parts of the human body have different sensing frequencies (Takahashi et al., 2018). Most existing haptic devices can only work for palm sensation at around 200 Hz, but not for other human bodies. It requires alternative parameter settings for specific tasks. The latest study started to determine the tactile feedback effects on the hairy skin parts rather than just the palm of hands (Pittera et al., 2022). However, current airborne US systems are still limited by output pressure and spatial resolution compared with laser-based tactile feedback systems. The latest piezoelectric micromachined ultrasonic transducer could replace traditional transducers with a small size and low power consumption to, suggesting potential applications in US haptics (Liu et al., 2022).

### 3.2. Airborne ultrasound application in human–computer interaction

Ultrasound haptic devices have been applied in various HCI applications, such as communications and entertainment, immersive virtual reality (VR), augmented reality, and user interfaces. In human communications and entertainment, typical applications include touching screens, buttons or other interaction widgets in mid-air (Monnai et al., 2014; Harrington et al., 2018). By providing haptic stimuli and enabling reliable gesture feedback, mid-air ultrasonic tactile feedback could improve entertainment experiences (Ablart et al., 2017; Freeman et al., 2019). In conjunction with holographic displays or VR headsets, US haptics could also create virtual objects in mid-air, increasing the immersion of interaction with virtual objects (Hoshi et al., 2009). Moreover, the airborne haptic systems could be integrated with virtual reality modalities without the need to wear or hold any equipment, such as AirPiano and rhythm VR games (Hwang et al., 2017; Georgiou et al., 2018).

In the medical field, airborne US has been utilized as a tactile interface in medical training simulators. The system used a hexagonal parabolic array with 271 ultrasonic transducers to create a focal point, achieving tactile sensations in a medical simulator for the first time (Hung et al., 2014). A recent study combined a US haptics system with standard visual virtual reality to improve palpation training experience and outcome, the feeling of interaction was further emphasized with the US haptic system (Puértolas Bálint and Althoefer, 2018). Notably, mid-air haptic interfaces could present Braille characters with an average accuracy of around 90% for blinded participants, indicating a promising application for facilitating daily multisensory experiences of visually impaired and blind people (Paneva et al., 2020).

In the field of robotics and automobiles, novel haptic interfaces with cable-driven force feedback and ultrasonic tactile feedback was proposed, which could integrate both touchless and in contact force feedback systems. Advanced haptic rendering algorithms may facilitate robotic control in especially virtual environments (Fan et al., 2020, 2022). A multimodal mid-air US haptic feedback system reduces eyes-off-the-road time without affecting driving performance, suggesting the function role of haptic feedback in increasing driving safety (Shakeri et al., 2018). Similarly, it was shown that gesture interface with US-based haptic feedback was particularly effective in decreasing visual demand (Large et al., 2019), and it can also be used for in-vehicle infotainment systems as a practical interaction language.

Despite these promising applications, ultrasonic-based in human-machine interaction is still in its preparatory stages, and future efforts are required to translate the airborne US into a practical interaction technique. Major changes for airborne US-based HCI include limited hardware performance, poor mid-air haptic rending and sensation techniques, and primitive integrations in specific tasks. Firstly, the hardware design of airborne pFUS should be further improved to enhance working efficiency and induce better tactile perceptions, such as the design of US transducers, the cost, and integration with other sensors, etc. Secondly, the mid-air haptic rendering algorithms could influence the properties of acoustic focus and vibration sensations, and the relationship between physical stimuli and the perception sensations induced should be quantified with psychophysics studies. Lastly, the user-experience of airborne US stimulation should be carefully optimized for specific applications (VR/AR games, training and simulation, and robotics), the functional role of pFUS in HCI should be further clarified.

## 4. Summary and future directions

Focused ultrasound stimulation is a non-invasive neuromodulation modality with high-resolution. Central focused ultrasound stimulation has gained wide attention in recent years. In contrast, peripheral focused ultrasound stimulation re-emerged in recently several years, despite having been investigated more than 60 years ago. Many technical and physiological questions about pFUS remain unanswered. Depending on the acoustic parameter settings and the transmission medium, pFUS could be classified as body-coupled contact US stimulation, commonly used for therapeutics or neuromodulation, and air-coupled contactless US systems, which enable various haptic HCI paradigms.

For body-coupled pFUS, low-intensity pFUS could be used for neuromodulation on nerve fibers, sub-organs and nerve endings. However, there is still no consensus regarding the precise working mechanisms, owing primarily to the non-thermal effects of the US. Most studies on pFUS of nerve fibers have been conducted on somatic PNS in animals, with preliminary trials in healthy humans were still in the early stage. Later translational studies in patients are still scarce. The latest pFUS research on sub-organs (autonomic PNS) for modulating the cholinergic anti-inflammatory and blood glucose pathways showed that pFUS could be a new bioelectronic medicine technique. However, previous studies were limited by restricted quantitative assessment tools for peripheral nerves, making it difficult to capture real-time neuronal functions and understand essential neuromodulation mechanisms. Further studies with improved quantitative assessment tools and reliable functional and structural patterns are necessary. Image guidance can provide precise localization of the stimulation targets, allowing for navigated pFUS treatments and functional imaging. Possible imaging techniques include US imaging, MRI, and optical techniques (Hynynen and Jones, 2016). Additionally, the tradeoff between high spatial resolution and low energy dissipation in transmission of US is still challenging. The improvements in US transducers and the optimizations of control systems are required to allow multi-locus and high-resolution system designs. As summarized in the previous review paper, broadband phased arrays, advanced focusing techniques, and complex acoustic modeling techniques could further improve US stimulation efficiency (Tyler et al., 2018). For therapeutics applications, current pFUS studies mainly focused on pain management and sub-organ modulation, but clinical studies in human patients were still scarce. Future large-sample randomized studies were required to translate these new techniques. Clinical applications in other symptoms are also appealing opportunities, the combinations with other neuromodulation techniques such as peripheral electrical or magnetic stimulation are also worth investigating. Safety issues are important, and suitable parameter settings are required for especially high-intensity applications.

Airborne US stimulation is an alternative modality for haptic human-computer interaction (HCI), but its resolution is less than that of body-coupled US stimulation paradigms. Current commercial mid-air US haptic systems could already serve as promising interaction techniques in specific HCI scenarios, such as gesture interactions, medical training, and robotics systems. However, current systems are still struggling to build complex tactile perceptions and achieve bionic stimulus, and US haptics remains in its infancy. The inherent problem of practicality and haptic output quality are the top concerns. Better user experiences and immersive applications are required, and the working mechanisms of airborne US stimulation on haptic perception should be better elaborated. Future studies should focus on improving the spatial and temporal perception of airborne US systems through optimized hardware and software designs, advanced haptic rendering methods and stronger sensations should be provided to expedite reliable interactions in different body positions. For airborne US for HCI applications, we should clarify the functional role of US in HCI and promote more reliable interaction paradigms.

## 5. Conclusion

This review provided an overview of peripheral focused ultrasound neurostimulation and its potential applications in the therapeutics and human-computer interaction. pFUS could enhance or inhibit neuronal functions depending on the acoustic parameters. Though the underlying working mechanisms of pFUS are not fully understood, its practical applications are emerging and worth further investigation. For therapeutic applications, technical improvements and large-sample clinical trials are required to validate the efficacy and safety of these state of art techniques in treating various diseases. Considering airborne US for human-computer interaction, touchless US systems are in the preliminary stage, and task-oriented US applications are needed to develop this promising interaction tool in the near future.

## Author contributions

S-CB and FL designed the review. S-CB, FL, and YX wrote the manuscript. YX, FL, LN, and HZ: critical reading of the manuscript. All authors contributed to the article and approved the submitted version.

## Funding

This study was supported by the National Key R&D Program of China (No. 2021YFF0501600), National Natural Science Foundation of China (No. 62101546), and Shenzhen Science and Technology Program (No. RCBS20210609104358078).

## Conflict of interest

The authors declare that the research was conducted in the absence of any commercial or financial relationships that could be construed as a potential conflict of interest.

## Publisher’s note

All claims expressed in this article are solely those of the authors and do not necessarily represent those of their affiliated organizations, or those of the publisher, the editors and the reviewers. Any product that may be evaluated in this article, or claim that may be made by its manufacturer, is not guaranteed or endorsed by the publisher.
